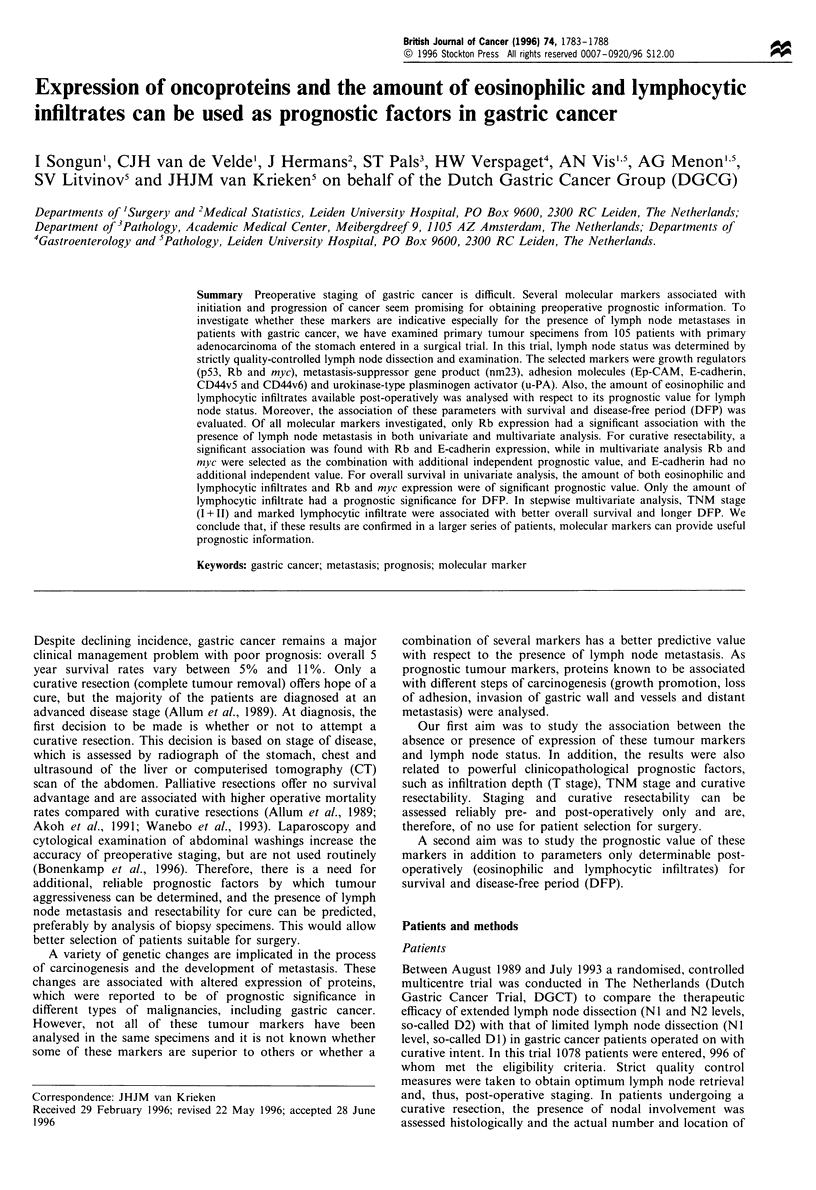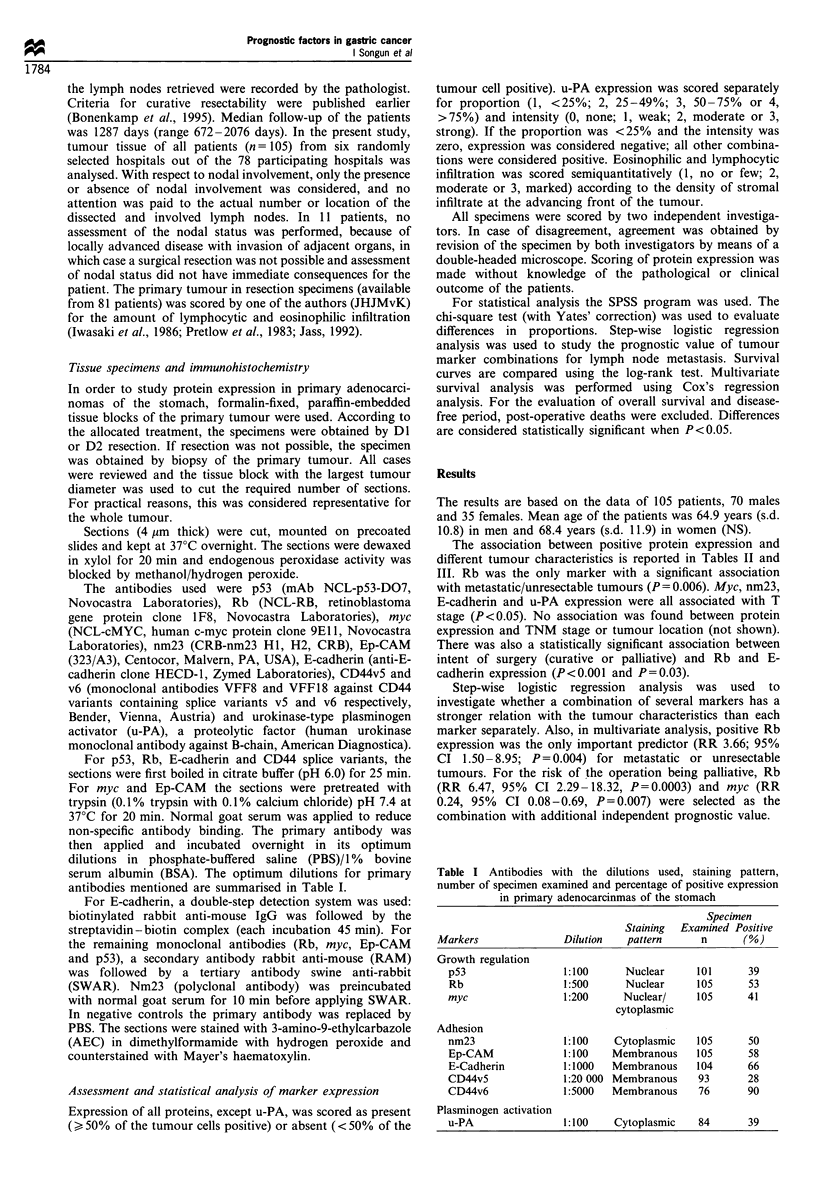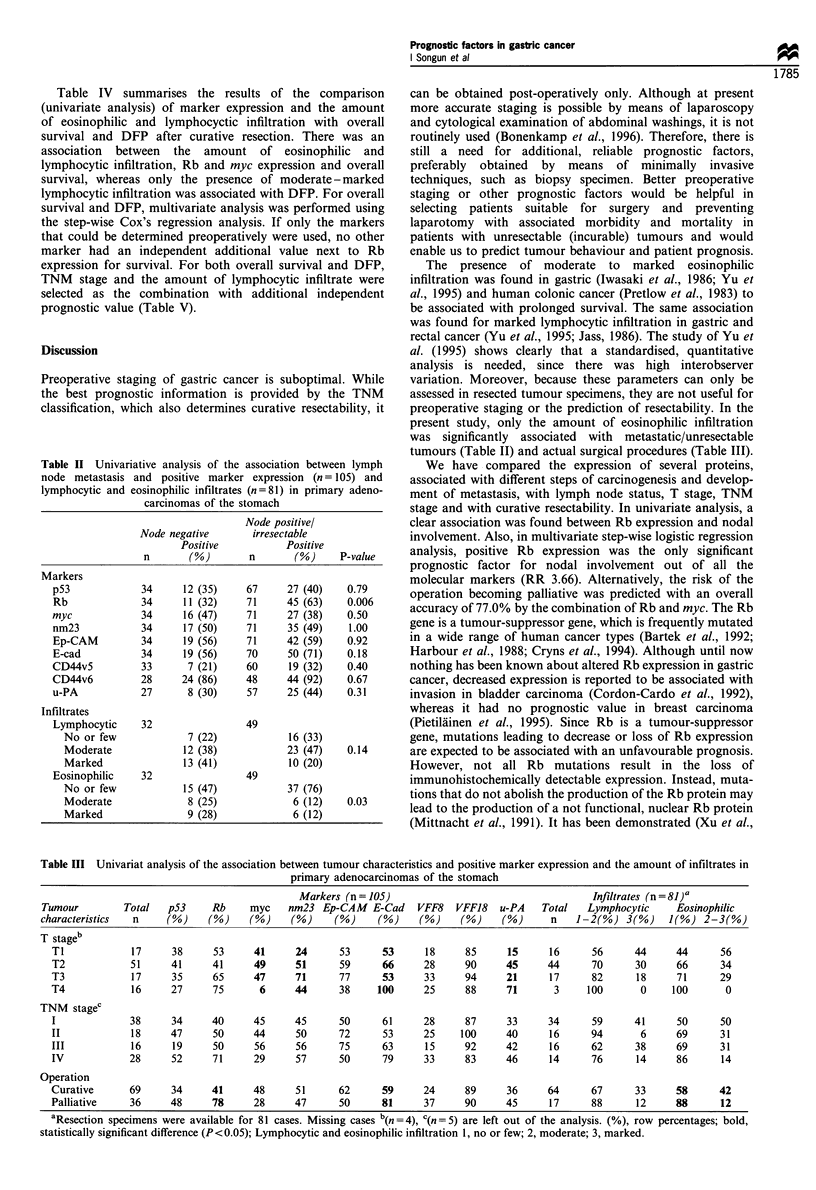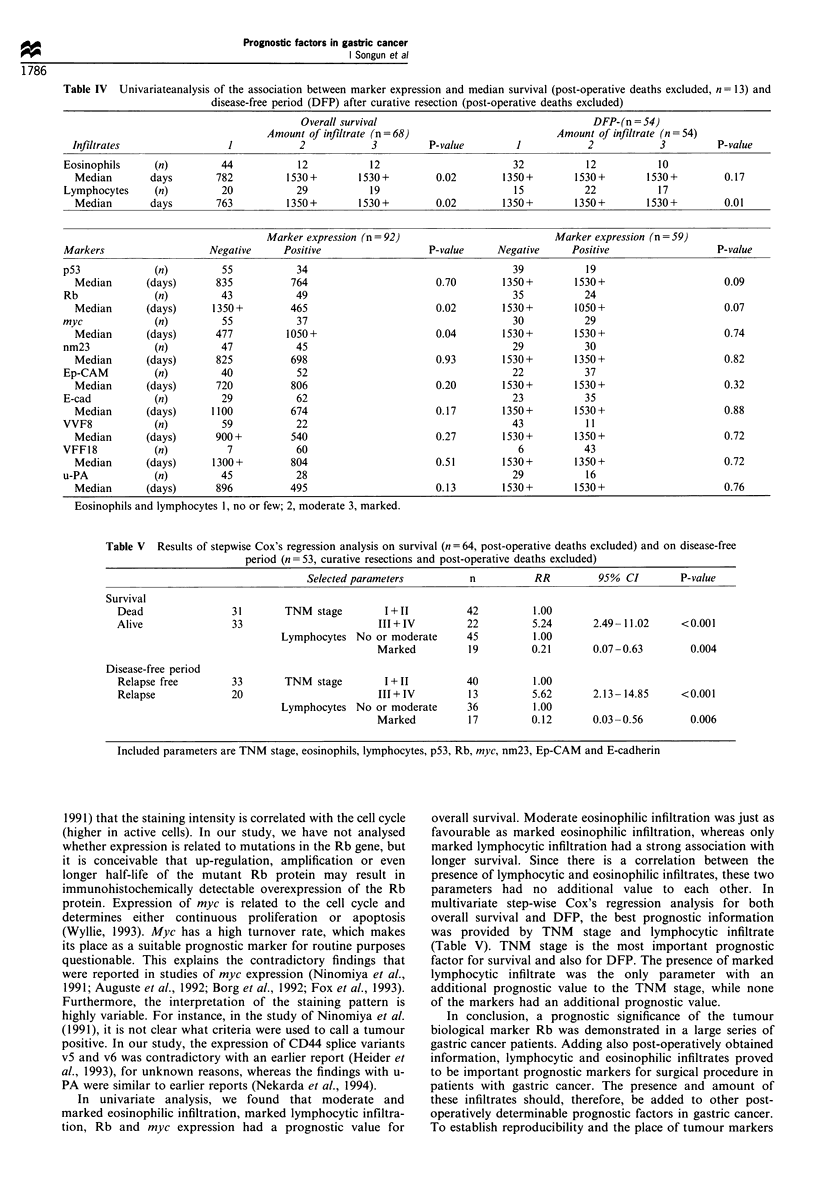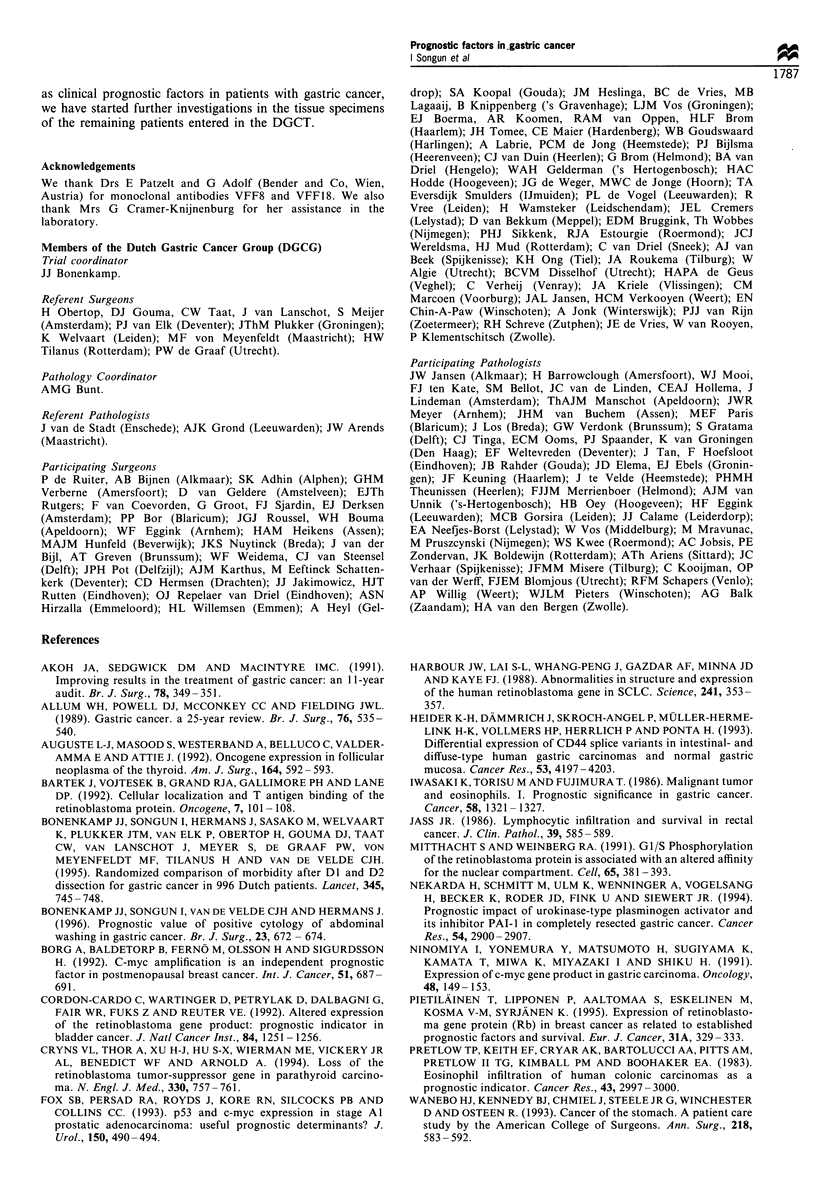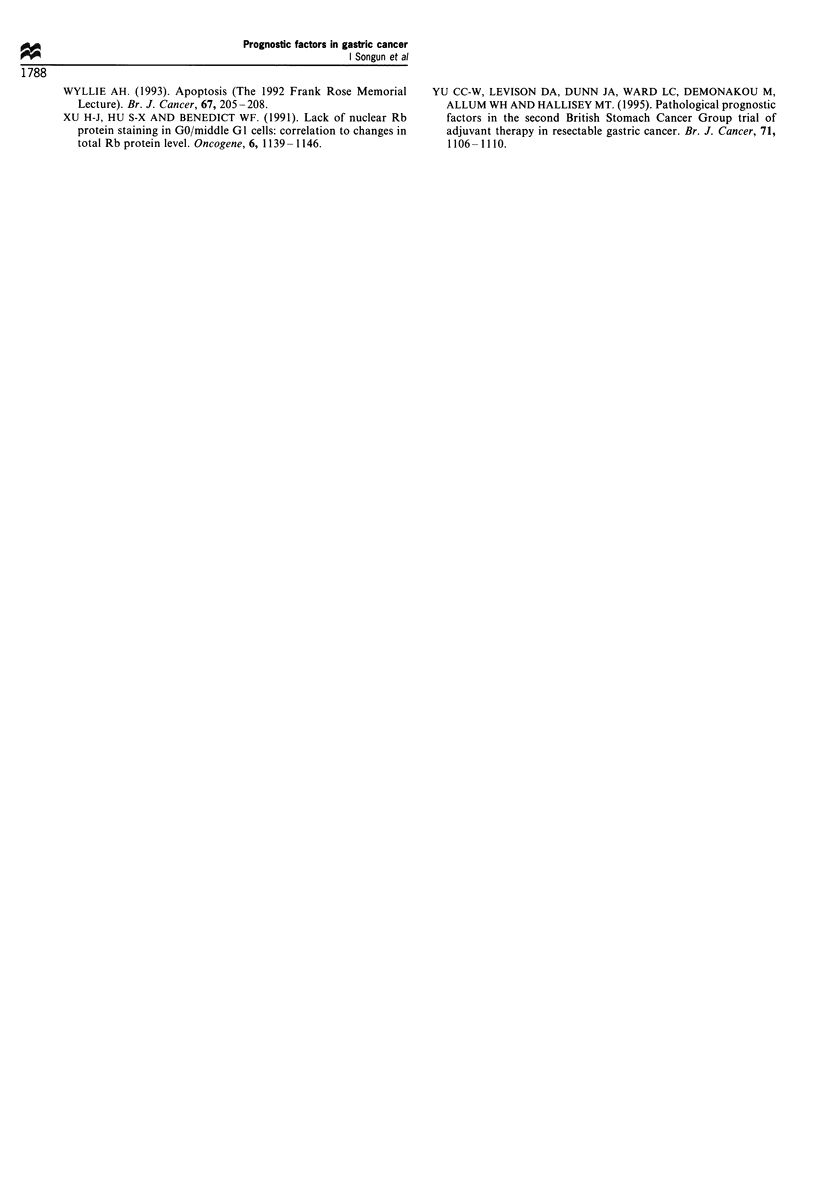# Expression of oncoproteins and the amount of eosinophilic and lymphocytic infiltrates can be used as prognostic factors in gastric cancer. Dutch Gastric Cancer Group (DGCG).

**DOI:** 10.1038/bjc.1996.630

**Published:** 1996-12

**Authors:** I. Songun, C. J. van de Velde, J. Hermans, S. T. Pals, H. W. Verspaget, A. N. Vis, A. G. Menon, S. V. Litvinov, J. H. van Krieken

**Affiliations:** Department of Surgery, Leiden University Hospital, The Netherlands.

## Abstract

Preoperative staging of gastric cancer is difficult. Several molecular markers associated with initiation and progression of cancer seem promising for obtaining preoperative prognostic information. To investigate whether these markers are indicative especially for the presence of lymph node metastases in patients with gastric cancer, we have examined primary tumour specimens from 105 patients with primary adenocarcinoma of the stomach entered in a surgical trial. In this trial, lymph node status was determined by strictly quality-controlled lymph node dissection and examination. The selected markers were growth regulators (p53, Rb and myc), metastasis-suppressor gene product (nm23), adhesion molecules (Ep-CAM, E-cadherin, CD44v5 and CD44v6) and urokinase-type plasminogen activator (u-PA). Also, the amount of eosinophilic and lymphocytic infiltrates available post-operatively was analysed with respect to its prognostic value for lymph node status. Moreover, the association of these parameters with survival and disease-free period (DFP) was evaluated. Of all molecular markers investigated, only Rb expression had a significant association with the presence of lymph node metastasis in both univariate and multivariate analysis. For curative resectability, a significant association was found with Rb and E-cadherin expression, while in multivariate analysis Rb and myc were selected as the combination with additional independent prognostic value, and E-cadherin had no additional independent value. For overall survival in univariate analysis, the amount of both eosinophilic and lymphocytic infiltrates and Rb and myc expression were of significant prognostic value. Only the amount of lymphocytic infiltrate had a prognostic significance for DFP. In stepwise multivariate analysis, TNM stage (I + II) and marked lymphocytic infiltrate were associated with better overall survival and longer DFP. We conclude that, if these results are confirmed in a larger series of patients, molecular markers can provide useful prognostic information.


British Journal of Cancer (1996) 74, 1783-1788
© 1996 Stockton Press All rights reserved 0007-0920/96 $12.00

### Expression of oncoproteins and the amount of eosinophilic and lymphocytic infiltrates can be used as prognostic factors in gastric cancer

I Songun[1], CJH van de Velde[1], J Hermans[2], ST Pals[3], HW Verspaget[4], AN Vis[1,5], AG Menon[1,5], SV Litvinov[5] and JHJM van Krieken[5] on behalf of the Dutch Gastric Cancer Group (DGCG)

*Departments of [1]Surgery and [2]Medical Statistics, Leiden University Hospital, PO Box 9600, 2300 RC Leiden, The Netherlands; Department of [3]Pathology, Academic Medical Center, Meibergdreef 9, 1105 AZ Amsterdam, The Netherlands; Departments of [4]Gastroenterology and [5]Pathology, Leiden University Hospital, PO Box 9600, 2300 RC Leiden, The Netherlands.*

**Summary** Preoperative staging of gastric cancer is difficult. Several molecular markers associated with initiation and progression of cancer seem promising for obtaining preoperative prognostic information. To investigate whether these markers are indicative especially for the presence of lymph node metastases in patients with gastric cancer, we have examined primary tumour specimens from 105 patients with primary adenocarcinoma of the stomach entered in a surgical trial. In this trial, lymph node status was determined by strictly quality-controlled lymph node dissection and examination. The selected markers were growth regulators (p53, Rb and *myc*), metastasis-suppressor gene product (nm23), adhesion molecules (Ep-CAM, E-cadherin, CD44v5 and CD44v6) and urokinase-type plasminogen activator (u-PA). Also, the amount of eosinophilic and lymphocytic infiltrates available post-operatively was analysed with respect to its prognostic value for lymph node status. Moreover, the association of these parameters with survival and disease-free period (DFP) was evaluated. Of all molecular markers investigated, only Rb expression had a significant association with the presence of lymph node metastasis in both univariate and multivariate analysis. For curative resectability, a significant association was found with Rb and E-cadherin expression, while in multivariate analysis Rb and *myc* were selected as the combination with additional independent prognostic value, and E-cadherin had no additional independent value. For overall survival in univariate analysis, the amount of both eosinophilic and lymphocytic infiltrates and Rb and *myc* expression were of significant prognostic value. Only the amount of lymphocytic infiltrate had a prognostic significance for DFP. In stepwise multivariate analysis, TNM stage (I+II) and marked lymphocytic infiltrate were associated with better overall survival and longer DFP. We conclude that, if these results are confirmed in a larger series of patients, molecular markers can provide useful prognostic information.

**Keywords:** gastric cancer; metastasis; prognosis; molecular marker

Despite declining incidence, gastric cancer remains a major clinical management problem with poor prognosis: overall 5 year survival rates vary between 5% and 11%. Only a curative resection (complete tumour removal) offers hope of a cure, but the majority of the patients are diagnosed at an advanced disease stage (Allum *et al.*, 1989). At diagnosis, the first decision to be made is whether or not to attempt a curative resection. This decision is based on stage of disease, which is assessed by radiograph of the stomach, chest and ultrasound of the liver or computerised tomography (CT) scan of the abdomen. Palliative resections offer no survival advantage and are associated with higher operative mortality rates compared with curative resections (Allum *et al.*, 1989; Akoh *et al.*, 1991; Wanebo *et al.*, 1993). Laparoscopy and cytological examination of abdominal washings increase the accuracy of preoperative staging, but are not used routinely (Bonenkamp *et al.*, 1996). Therefore, there is a need for additional, reliable prognostic factors by which tumour aggressiveness can be determined, and the presence of lymph node metastasis and resectability for cure can be predicted, preferably by analysis of biopsy specimens. This would allow better selection of patients suitable for surgery.

A variety of genetic changes are implicated in the process of carcinogenesis and the development of metastasis. These changes are associated with altered expression of proteins, which were reported to be of prognostic significance in different types of malignancies, including gastric cancer. However, not all of these tumour markers have been analysed in the same specimens and it is not known whether some of these markers are superior to others or whether a combination of several markers has a better predictive value with respect to the presence of lymph node metastasis. As prognostic tumour markers, proteins known to be associated with different steps of carcinogenesis (growth promotion, loss of adhesion, invasion of gastric wall and vessels and distant metastasis) were analysed.

Our first aim was to study the association between the absence or presence of expression of these tumour markers and lymph node status. In addition, the results were also related to powerful clinicopathological prognostic factors, such as infiltration depth (T stage), TNM stage and curative resectability. Staging and curative resectability can be assessed reliably pre- and post-operatively only and are, therefore, of no use for patient selection for surgery.

A second aim was to study the prognostic value of these markers in addition to parameters only determinable post-operatively (eosinophilic and lymphocytic infiltrates) for survival and disease-free period (DFP).

### Patients and methods

### *Patients*

Between August 1989 and July 1993 a randomised, controlled multicentre trial was conducted in The Netherlands (Dutch Gastric Cancer Trial, DGCT) to compare the therapeutic efficacy of extended lymph node dissection (N1 and N2 levels, so-called D2) with that of limited lymph node dissection (N1 level, so-called D1) in gastric cancer patients operated on with curative intent. In this trial 1078 patients were entered, 996 of whom met the eligibility criteria. Strict quality control measures were taken to obtain optimum lymph node retrieval and, thus, post-operative staging. In patients undergoing a curative resection, the presence of nodal involvement was assessed histologically and the actual number and location of

Correspondence: JHJM van Krieken
Received 29 February 1996; revised 22 May 1996; accepted 28 June 1996

the lymph nodes retrieved were recorded by the pathologist. Criteria for curative resectability were published earlier (Bonenkamp *et al*., 1995). Median follow-up of the patients was 1287 days (range 672–2076 days). In the present study, tumour tissue of all patients ($n = 105$) from six randomly selected hospitals out of the 78 participating hospitals was analysed. With respect to nodal involvement, only the presence or absence of nodal involvement was considered, and no attention was paid to the actual number or location of the dissected and involved lymph nodes. In 11 patients, no assessment of the nodal status was performed, because of locally advanced disease with invasion of adjacent organs, in which case a surgical resection was not possible and assessment of nodal status did not have immediate consequences for the patient. The primary tumour in resection specimens (available from 81 patients) was scored by one of the authors (JHJMvK) for the amount of lymphocytic and eosinophilic infiltration (Iwasaki *et al*., 1986; Pretlow *et al*., 1983; Jass, 1992).

### *Tissue specimens and immunohistochemistry*

In order to study protein expression in primary adenocarcinomas of the stomach, formalin-fixed, paraffin-embedded tissue blocks of the primary tumour were used. According to the allocated treatment, the specimens were obtained by D1 or D2 resection. If resection was not possible, the specimen was obtained by biopsy of the primary tumour. All cases were reviewed and the tissue block with the largest tumour diameter was used to cut the required number of sections. For practical reasons, this was considered representative for the whole tumour.

Sections (4 μm thick) were cut, mounted on precoated slides and kept at 37°C overnight. The sections were dewaxed in xylol for 20 min and endogenous peroxidase activity was blocked by methanol/hydrogen peroxide.

The antibodies used were p53 (mAb NCL-p53-DO7, Novocastra Laboratories), Rb (NCL-RB, retinoblastoma gene protein clone 1F8, Novocastra Laboratories), *myc* (NCL-cMYC, human c-myc protein clone 9E11, Novocastra Laboratories), nm23 (CRB-nm23 H1, H2, CRB), Ep-CAM (323/A3), Centocor, Malvern, PA, USA), E-cadherin (anti-E-cadherin clone HECD-1, Zymed Laboratories), CD44v5 and v6 (monoclonal antibodies VFF8 and VFF18 against CD44 variants containing splice variants v5 and v6 respectively, Bender, Vienna, Austria) and urokinase-type plasminogen activator (u-PA), a proteolytic factor (human urokinase monoclonal antibody against B-chain, American Diagnostica).

For p53, Rb, E-cadherin and CD44 splice variants, the sections were first boiled in citrate buffer (pH 6.0) for 25 min. For *myc* and Ep-CAM the sections were pretreated with trypsin (0.1% trypsin with 0.1% calcium chloride) pH 7.4 at 37°C for 20 min. Normal goat serum was applied to reduce non-specific antibody binding. The primary antibody was then applied and incubated overnight in its optimum dilutions in phosphate-buffered saline (PBS)/1% bovine serum albumin (BSA). The optimum dilutions for primary antibodies mentioned are summarised in Table I.

For E-cadherin, a double-step detection system was used: biotinylated rabbit anti-mouse IgG was followed by the streptavidin–biotin complex (each incubation 45 min). For the remaining monoclonal antibodies (Rb, *myc*, Ep-CAM and p53), a secondary antibody rabbit anti-mouse (RAM) was followed by a tertiary antibody swine anti-rabbit (SWAR). Nm23 (polyclonal antibody) was preincubated with normal goat serum for 10 min before applying SWAR. In negative controls the primary antibody was replaced by PBS. The sections were stained with 3-amino-9-ethylcarbazole (AEC) in dimethylformamide with hydrogen peroxide and counterstained with Mayer's haematoxylin.

### *Assessment and statistical analysis of marker expression*

Expression of all proteins, except u-PA, was scored as present (≥50% of the tumour cells positive) or absent (<50% of the tumour cell positive). u-PA expression was scored separately for proportion (1, <25%; 2, 25–49%; 3, 50–75% or 4, >75%) and intensity (0, none; 1, weak; 2, moderate or 3, strong). If the proportion was <25% and the intensity was zero, expression was considered negative; all other combinations were considered positive. Eosinophilic and lymphocytic infiltration was scored semiquantitatively (1, no or few; 2, moderate or 3, marked) according to the density of stromal infiltrate at the advancing front of the tumour.

All specimens were scored by two independent investigators. In case of disagreement, agreement was obtained by revision of the specimen by both investigators by means of a double-headed microscope. Scoring of protein expression was made without knowledge of the pathological or clinical outcome of the patients.

For statistical analysis the SPSS program was used. The chi-square test (with Yates' correction) was used to evaluate differences in proportions. Step-wise logistic regression analysis was used to study the prognostic value of tumour marker combinations for lymph node metastasis. Survival curves are compared using the log-rank test. Multivariate survival analysis was performed using Cox's regression analysis. For the evaluation of overall survival and disease-free period, post-operative deaths were excluded. Differences are considered statistically significant when $P < 0.05$.

### Results

The results are based on the data of 105 patients, 70 males and 35 females. Mean age of the patients was 64.9 years (s.d. 10.8) in men and 68.4 years (s.d. 11.9) in women (NS).

The association between positive protein expression and different tumour characteristics is reported in Tables II and III. Rb was the only marker with a significant association with metastatic/unresectable tumours ($P = 0.006$). *Myc*, nm23, E-cadherin and u-PA expression were all associated with T stage ($P < 0.05$). No association was found between protein expression and TNM stage or tumour location (not shown). There was also a statistically significant association between intent of surgery (curative or palliative) and Rb and E-cadherin expression ($P < 0.001$ and $P = 0.03$).

Step-wise logistic regression analysis was used to investigate whether a combination of several markers has a stronger relation with the tumour characteristics than each marker separately. Also, in multivariate analysis, positive Rb expression was the only important predictor (RR 3.66; 95% CI 1.50–8.95; $P = 0.004$) for metastatic or unresectable tumours. For the risk of the operation being palliative, Rb (RR 6.47, 95% CI 2.29–18.32, $P = 0.0003$) and *myc* (RR 0.24, 95% CI 0.08–0.69, $P = 0.007$) were selected as the combination with additional independent prognostic value.

**Table I** Antibodies with the dilutions used, staining pattern, number of specimen examined and percentage of positive expression in primary adenocarcinmas of the stomach

| *Markers* | *Dilution* | *Staining pattern* | *Specimen Examined* n | *Specimen Positive (%)* |
|---|---|---|---|---|
| Growth regulation | | | | |
| p53 | 1:100 | Nuclear | 101 | 39 |
| Rb | 1:500 | Nuclear | 105 | 53 |
| *myc* | 1:200 | Nuclear/ cytoplasmic | 105 | 41 |
| Adhesion | | | | |
| nm23 | 1:100 | Cytoplasmic | 105 | 50 |
| Ep-CAM | 1:100 | Membranous | 105 | 58 |
| E-Cadherin | 1:1000 | Membranous | 104 | 66 |
| CD44v5 | 1:20 000 | Membranous | 93 | 28 |
| CD44v6 | 1:5000 | Membranous | 76 | 90 |
| Plasminogen activation | | | | |
| u-PA | 1:100 | Cytoplasmic | 84 | 39 |

Table IV summarises the results of the comparison (univariate analysis) of marker expression and the amount of eosinophilic and lymphocyctic infiltration with overall survival and DFP after curative resection. There was an association between the amount of eosinophilic and lymphocytic infiltration, Rb and *myc* expression and overall survival, whereas only the presence of moderate–marked lymphocytic infiltration was associated with DFP. For overall survival and DFP, multivariate analysis was performed using the step-wise Cox's regression analysis. If only the markers that could be determined preoperatively were used, no other marker had an independent additional value next to Rb expression for survival. For both overall survival and DFP, TNM stage and the amount of lymphocytic infiltrate were selected as the combination with additional independent prognostic value (Table V).

### Discussion

Preoperative staging of gastric cancer is suboptimal. While the best prognostic information is provided by the TNM classification, which also determines curative resectability, it can be obtained post-operatively only. Although at present more accurate staging is possible by means of laparoscopy and cytological examination of abdominal washings, it is not routinely used (Bonenkamp *et al.*, 1996). Therefore, there is still a need for additional, reliable prognostic factors, preferably obtained by means of minimally invasive techniques, such as biopsy specimen. Better preoperative staging or other prognostic factors would be helpful in selecting patients suitable for surgery and preventing laparotomy with associated morbidity and mortality in patients with unresectable (incurable) tumours and would enable us to predict tumour behaviour and patient prognosis.

The presence of moderate to marked eosinophilic infiltration was found in gastric (Iwasaki *et al.*, 1986; Yu *et al.*, 1995) and human colonic cancer (Pretlow *et al.*, 1983) to be associated with prolonged survival. The same association was found for marked lymphocytic infiltration in gastric and rectal cancer (Yu *et al.*, 1995; Jass, 1986). The study of Yu *et al.* (1995) shows clearly that a standardised, quantitative analysis is needed, since there was high interobserver variation. Moreover, because these parameters can only be assessed in resected tumour specimens, they are not useful for preoperative staging or the prediction of resectability. In the present study, only the amount of eosinophilic infiltration was significantly associated with metastatic/unresectable tumours (Table II) and actual surgical procedures (Table III).

We have compared the expression of several proteins, associated with different steps of carcinogenesis and development of metastasis, with lymph node status, T stage, TNM stage and with curative resectability. In univariate analysis, a clear association was found between Rb expression and nodal involvement. Also, in multivariate step-wise logistic regression analysis, positive Rb expression was the only significant prognostic factor for nodal involvement out of all the molecular markers (RR 3.66). Alternatively, the risk of the operation becoming palliative was predicted with an overall accuracy of 77.0% by the combination of Rb and *myc*. The Rb gene is a tumour-suppressor gene, which is frequently mutated in a wide range of human cancer types (Bartek *et al.*, 1992; Harbour *et al.*, 1988; Cryns *et al.*, 1994). Although until now nothing has been known about altered Rb expression in gastric cancer, decreased expression is reported to be associated with invasion in bladder carcinoma (Cordon-Cardo *et al.*, 1992), whereas it had no prognostic value in breast carcinoma (Pietiläinen *et al.*, 1995). Since Rb is a tumour-suppressor gene, mutations leading to decrease or loss of Rb expression are expected to be associated with an unfavourable prognosis. However, not all Rb mutations result in the loss of immunohistochemically detectable expression. Instead, mutations that do not abolish the production of the Rb protein may lead to the production of a not functional, nuclear Rb protein (Mittnacht *et al.*, 1991). It has been demonstrated (Xu *et al.*,

**Table II** Univariative analysis of the association between lymph node metastasis and positive marker expression ($n=105$) and lymphocytic and eosinophilic infiltrates ($n=81$) in primary adenocarcinomas of the stomach

| | *Node negative* n | *Node negative* *Positive (%)* | *Node positive/ irresectable* n | *Node positive/ irresectable* *Positive (%)* | P-*value* |
|---|---|---|---|---|---|
| Markers | | | | | |
| p53 | 34 | 12 (35) | 67 | 27 (40) | 0.79 |
| Rb | 34 | 11 (32) | 71 | 45 (63) | 0.006 |
| *myc* | 34 | 16 (47) | 71 | 27 (38) | 0.50 |
| nm23 | 34 | 17 (50) | 71 | 35 (49) | 1.00 |
| Ep-CAM | 34 | 19 (56) | 71 | 42 (59) | 0.92 |
| E-cad | 34 | 19 (56) | 70 | 50 (71) | 0.18 |
| CD44v5 | 33 | 7 (21) | 60 | 19 (32) | 0.40 |
| CD44v6 | 28 | 24 (86) | 48 | 44 (92) | 0.67 |
| u-PA | 27 | 8 (30) | 57 | 25 (44) | 0.31 |
| Infiltrates | | | | | |
| Lymphocytic | 32 | | 49 | | |
| No or few | | 7 (22) | | 16 (33) | |
| Moderate | | 12 (38) | | 23 (47) | 0.14 |
| Marked | | 13 (41) | | 10 (20) | |
| Eosinophilic | 32 | | 49 | | |
| No or few | | 15 (47) | | 37 (76) | |
| Moderate | | 8 (25) | | 6 (12) | 0.03 |
| Marked | | 9 (28) | | 6 (12) | |

**Table III** Univariat analysis of the association between tumour characteristics and positive marker expression and the amount of infiltrates in primary adenocarcinomas of the stomach

| *Tumour characteristics* | *Total* n | *p53 (%)* | *Rb (%)* | myc *(%)* | *Markers* (n=*105*) *nm23 (%)* | *Ep-CAM (%)* | *E-Cad (%)* | *VFF8 (%)* | *VFF18 (%)* | *u-PA (%)* | *Total* n | *Infiltrates* (n=*81*)[a] *Lymphocytic 1–2(%)* | *Lymphocytic 3(%)* | *Eosinophilic 1(%)* | *Eosinophilic 2–3(%)* |
|---|---|---|---|---|---|---|---|---|---|---|---|---|---|---|---|
| T stage[b] | | | | | | | | | | | | | | | |
| T1 | 17 | 38 | 53 | **41** | **24** | 53 | **53** | 18 | 85 | **15** | 16 | 56 | 44 | 44 | 56 |
| T2 | 51 | 41 | 41 | **49** | **51** | 59 | **66** | 28 | 90 | **45** | 44 | 70 | 30 | 66 | 34 |
| T3 | 17 | 35 | 65 | **47** | **71** | 77 | **53** | 33 | 94 | **21** | 17 | 82 | 18 | 71 | 29 |
| T4 | 16 | 27 | 75 | **6** | **44** | 38 | **100** | 25 | 88 | **71** | 3 | 100 | 0 | 100 | 0 |
| TNM stage[c] | | | | | | | | | | | | | | | |
| I | 38 | 34 | 40 | 45 | 45 | 50 | 61 | 28 | 87 | 33 | 34 | 59 | 41 | 50 | 50 |
| II | 18 | 47 | 50 | 44 | 50 | 72 | 53 | 25 | 100 | 40 | 16 | 94 | 6 | 69 | 31 |
| III | 16 | 19 | 50 | 56 | 56 | 75 | 63 | 15 | 92 | 42 | 16 | 62 | 38 | 69 | 31 |
| IV | 28 | 52 | 71 | 29 | 57 | 50 | 79 | 33 | 83 | 46 | 14 | 76 | 14 | 86 | 14 |
| Operation | | | | | | | | | | | | | | | |
| Curative | 69 | 34 | **41** | 48 | 51 | 62 | **59** | 24 | 89 | 36 | 64 | 67 | 33 | **58** | **42** |
| Palliative | 36 | 48 | **78** | 28 | 47 | 50 | **81** | 37 | 90 | 45 | 17 | 88 | 12 | **88** | **12** |

[a]Resection specimens were available for 81 cases. Missing cases [b]($n=4$), [c]($n=5$) are left out of the analysis. (%), row percentages; bold, statistically significant difference ($P<0.05$); Lymphocytic and eosinophilic infiltration 1, no or few; 2, moderate; 3, marked.

**Table IV** Univariateanalysis of the association between marker expression and median survival (post-operative deaths excluded, $n=13$) and disease-free period (DFP) after curative resection (post-operative deaths excluded)

| Infiltrates | | Overall survival Amount of infiltrate (n = 68) 1 | 2 | 3 | P-value | DFP-(n = 54) Amount of infiltrate (n = 54) 1 | 2 | 3 | P-value |
|---|---|---|---|---|---|---|---|---|---|
| Eosinophils | (n) | 44 | 12 | 12 | | 32 | 12 | 10 | |
| Median | days | 782 | 1530+ | 1530+ | 0.02 | 1350+ | 1530+ | 1530+ | 0.17 |
| Lymphocytes | (n) | 20 | 29 | 19 | | 15 | 22 | 17 | |
| Median | days | 763 | 1350+ | 1530+ | 0.02 | 1350+ | 1350+ | 1530+ | 0.01 |

| Markers | | Marker expression (n = 92) Negative | Positive | P-value | Marker expression (n = 59) Negative | Positive | P-value |
|---|---|---|---|---|---|---|---|
| p53 | (n) | 55 | 34 | | 39 | 19 | |
| Median | (days) | 835 | 764 | 0.70 | 1350+ | 1530+ | 0.09 |
| Rb | (n) | 43 | 49 | | 35 | 24 | |
| Median | (days) | 1350+ | 465 | 0.02 | 1530+ | 1050+ | 0.07 |
| *myc* | (n) | 55 | 37 | | 30 | 29 | |
| Median | (days) | 477 | 1050+ | 0.04 | 1530+ | 1530+ | 0.74 |
| nm23 | (n) | 47 | 45 | | 29 | 30 | |
| Median | (days) | 825 | 698 | 0.93 | 1530+ | 1350+ | 0.82 |
| Ep-CAM | (n) | 40 | 52 | | 22 | 37 | |
| Median | (days) | 720 | 806 | 0.20 | 1530+ | 1530+ | 0.32 |
| E-cad | (n) | 29 | 62 | | 23 | 35 | |
| Median | (days) | 1100 | 674 | 0.17 | 1350+ | 1530+ | 0.88 |
| VVF8 | (n) | 59 | 22 | | 43 | 11 | |
| Median | (days) | 900+ | 540 | 0.27 | 1530+ | 1350+ | 0.72 |
| VFF18 | (n) | 7 | 60 | | 6 | 43 | |
| Median | (days) | 1300+ | 804 | 0.51 | 1530+ | 1350+ | 0.72 |
| u-PA | (n) | 45 | 28 | | 29 | 16 | |
| Median | (days) | 896 | 495 | 0.13 | 1530+ | 1530+ | 0.76 |

Eosinophils and lymphocytes 1, no or few; 2, moderate 3, marked.

**Table V** Results of stepwise Cox's regression analysis on survival ($n=64$, post-operative deaths excluded) and on disease-free period ($n=53$, curative resections and post-operative deaths excluded)

| | | Selected parameters | | n | RR | 95% CI | P-value |
|---|---|---|---|---|---|---|---|
| Survival | | | | | | | |
| Dead | 31 | TNM stage | I+II | 42 | 1.00 | | |
| Alive | 33 | | III+IV | 22 | 5.24 | 2.49–11.02 | <0.001 |
| | | Lymphocytes | No or moderate | 45 | 1.00 | | |
| | | | Marked | 19 | 0.21 | 0.07–0.63 | 0.004 |
| Disease-free period | | | | | | | |
| Relapse free | 33 | TNM stage | I+II | 40 | 1.00 | | |
| Relapse | 20 | | III+IV | 13 | 5.62 | 2.13–14.85 | <0.001 |
| | | Lymphocytes | No or moderate | 36 | 1.00 | | |
| | | | Marked | 17 | 0.12 | 0.03–0.56 | 0.006 |

Included parameters are TNM stage, eosinophils, lymphocytes, p53, Rb, *myc*, nm23, Ep-CAM and E-cadherin

1991) that the staining intensity is correlated with the cell cycle (higher in active cells). In our study, we have not analysed whether expression is related to mutations in the Rb gene, but it is conceivable that up-regulation, amplification or even longer half-life of the mutant Rb protein may result in immunohistochemically detectable overexpression of the Rb protein. Expression of *myc* is related to the cell cycle and determines either continuous proliferation or apoptosis (Wyllie, 1993). *Myc* has a high turnover rate, which makes its place as a suitable prognostic marker for routine purposes questionable. This explains the contradictory findings that were reported in studies of *myc* expression (Ninomiya *et al.*, 1991; Auguste *et al.*, 1992; Borg *et al.*, 1992; Fox *et al.*, 1993). Furthermore, the interpretation of the staining pattern is highly variable. For instance, in the study of Ninomiya *et al.* (1991), it is not clear what criteria were used to call a tumour positive. In our study, the expression of CD44 splice variants v5 and v6 was contradictory with an earlier report (Heider *et al.*, 1993), for unknown reasons, whereas the findings with u-PA were similar to earlier reports (Nekarda *et al.*, 1994).

In univariate analysis, we found that moderate and marked eosinophilic infiltration, marked lymphocytic infiltration, Rb and *myc* expression had a prognostic value for overall survival. Moderate eosinophilic infiltration was just as favourable as marked eosinophilic infiltration, whereas only marked lymphocytic infiltration had a strong association with longer survival. Since there is a correlation between the presence of lymphocytic and eosinophilic infiltrates, these two parameters had no additional value to each other. In multivariate step-wise Cox's regression analysis for both overall survival and DFP, the best prognostic information was provided by TNM stage and lymphocytic infiltrate (Table V). TNM stage is the most important prognostic factor for survival and also for DFP. The presence of marked lymphocytic infiltrate was the only parameter with an additional prognostic value to the TNM stage, while none of the markers had an additional prognostic value.

In conclusion, a prognostic significance of the tumour biological marker Rb was demonstrated in a large series of gastric cancer patients. Adding also post-operatively obtained information, lymphocytic and eosinophilic infiltrates proved to be important prognostic markers for surgical procedure in patients with gastric cancer. The presence and amount of these infiltrates should, therefore, be added to other post-operatively determinable prognostic factors in gastric cancer. To establish reproducibility and the place of tumour markers as clinical prognostic factors in patients with gastric cancer, we have started further investigations in the tissue specimens of the remaining patients entered in the DGCT.

**Acknowledgements**

We thank Drs E Patzelt and G Adolf (Bender and Co, Wien, Austria) for monoclonal antibodies VFF8 and VFF18. We also thank Mrs G Cramer-Knijnenburg for her assistance in the laboratory.

**Members of the Dutch Gastric Cancer Group (DGCG)**

*Trial coordinator*
JJ Bonenkamp.

*Referent Surgeons*
H Obertop, DJ Gouma, CW Taat, J van Lanschot, S Meijer (Amsterdam); PJ van Elk (Deventer); JThM Plukker (Groningen); K Welvaart (Leiden); MF von Meyenfeldt (Maastricht); HW Tilanus (Rotterdam); PW de Graaf (Utrecht).

*Pathology Coordinator*
AMG Bunt.

*Referent Pathologists*
J van de Stadt (Enschede); AJK Grond (Leeuwarden); JW Arends (Maastricht).

*Participating Surgeons*
P de Ruiter, AB Bijnen (Alkmaar); SK Adhin (Alphen); GHM Verberne (Amersfoort); D van Geldere (Amstelveen); EJTh Rutgers; F van Coevorden, G Groot, FJ Sjardin, EJ Derksen (Amsterdam); PP Bor (Blaricum); JGJ Roussel, WH Bouma (Apeldoorn); WF Eggink (Arnhem); HAM Heikens (Assen); MAJM Hunfeld (Beverwijk); JKS Nuytinck (Breda); J van der Bijl, AT Greven (Brunssum); WF Weidema, CJ van Steensel (Delft); JPH Pot (Delfzijl); AJM Karthus, M Eeftinck Schattenkerk (Deventer); CD Hermsen (Drachten); JJ Jakimowicz, HJT Rutten (Eindhoven); OJ Repelaer van Driel (Eindhoven); ASN Hirzalla (Emmeloord); HL Willemsen (Emmen); A Heyl (Geldrop); SA Koopal (Gouda); JM Heslinga, BC de Vries, MB Lagaaij, B Knippenberg ('s Gravenhage); LJM Vos (Groningen); EJ Boerma, AR Koomen, RAM van Oppen, HLF Brom (Haarlem); JH Tomee, CE Maier (Hardenberg); WB Goudswaard (Harlingen); A Labrie, PCM de Jong (Heemstede); PJ Bijlsma (Heerenveen); CJ van Duin (Heerlen); G Brom (Helmond); BA van Driel (Hengelo); WAH Gelderman ('s Hertogenbosch); HAC Hodde (Hoogeveen); JG de Weger, MWC de Jonge (Hoorn); TA Eversdijk Smulders (IJmuiden); PL de Vogel (Leeuwarden); R Vree (Leiden); H Wamsteker (Leidschendam); JEL Cremers (Lelystad); D van Bekkum (Meppel); EDM Bruggink, Th Wobbes (Nijmegen); PHJ Sikkenk, RJA Estourgie (Roermond); JCJ Wereldsma, HJ Mud (Rotterdam); C van Driel (Sneek); AJ van Beek (Spijkenisse); KH Ong (Tiel); JA Roukema (Tilburg); W Algie (Utrecht); BCVM Disselhof (Utrecht); HAPA de Geus (Veghel); C Verheij (Venray); JA Kriele (Vlissingen); CM Marcoen (Voorburg); JAL Jansen, HCM Verkooyen (Weert); EN Chin-A-Paw (Winschoten); A Jonk (Winterswijk); PJJ van Rijn (Zoetermeer); RH Schreve (Zutphen); JE de Vries, W van Rooyen, P Klementschitsch (Zwolle).

*Participating Pathologists*
JW Jansen (Alkmaar); H Barrowclough (Amersfoort), WJ Mooi, FJ ten Kate, SM Bellot, JC van de Linden, CEAJ Hollema, J Lindeman (Amsterdam); ThAJM Manschot (Apeldoorn); JWR Meyer (Arnhem); JHM van Buchem (Assen); MEF Paris (Blaricum); J Los (Breda); GW Verdonk (Brunssum); S Gratama (Delft); CJ Tinga, ECM Ooms, PJ Spaander, K van Groningen (Den Haag); EF Weltevreden (Deventer); J Tan, F Hoefsloot (Eindhoven); JB Rahder (Gouda); JD Elema, EJ Ebels (Groningen); JF Keuning (Haarlem); J te Velde (Heemstede); PHMH Theunissen (Heerlen); FJJM Merrienboer (Helmond); AJM van Unnik ('s-Hertogenbosch); HB Oey (Hoogeveen); HF Eggink (Leeuwarden); MCB Gorsira (Leiden); JJ Calame (Leiderdorp); EA Neefjes-Borst (Lelystad); W Vos (Middelburg); M Mravunac, M Pruszcynski (Nijmegen); WS Kwee (Roermond); AC Jobsis, PE Zondervan, JK Boldewijn (Rotterdam); ATh Ariens (Sittard); JC Verhaar (Spijkenisse); JFMM Misere (Tilburg); C Kooijman, OP van der Werff, FJEM Blomjous (Utrecht); RFM Schapers (Venlo); AP Willig (Weert); WJLM Pieters (Winschoten); AG Balk (Zaandam); HA van den Bergen (Zwolle).

### References

AKOH JA, SEDGWICK DM AND MACINTYRE IMC. (1991). Improving results in the treatment of gastric cancer: an 11-year audit. *Br. J. Surg.*, **78,** 349–351.

ALLUM WH, POWELL DJ, MCCONKEY CC AND FIELDING JWL. (1989). Gastric cancer. a 25-year review. *Br. J. Surg.*, **76,** 535–540.

AUGUSTE L-J, MASOOD S, WESTERBAND A, BELLUCO C, VALDERAMMA E AND ATTIE J. (1992). Oncogene expression in follicular neoplasma of the thyroid. *Am. J. Surg.*, **164,** 592–593.

BARTEK J, VOJTESEK B, GRAND RJA, GALLIMORE PH AND LANE DP. (1992). Cellular localization and T antigen binding of the retinoblastoma protein. *Oncogene*, **7,** 101–108.

BONENKAMP JJ, SONGUN I, HERMANS J, SASAKO M, WELVAART K, PLUKKER JTM, VAN ELK P, OBERTOP H, GOUMA DJ, TAAT CW, VAN LANSCHOT J, MEYER S, DE GRAAF PW, VON MEYENFELDT MF, TILANUS H AND VAN DE VELDE CJH. (1995). Randomized comparison of morbidity after D1 and D2 dissection for gastric cancer in 996 Dutch patients. *Lancet*, **345,** 745–748.

BONENKAMP JJ, SONGUN I, VAN DE VELDE CJH AND HERMANS J. (1996). Prognostic value of positive cytology of abdominal washing in gastric cancer. *Br. J. Surg.*, **23,** 672 – 674.

BORG A, BALDETORP B, FERNÖ M, OLSSON H AND SIGURDSSON H. (1992). C-myc amplification is an independent prognostic factor in postmenopausal breast cancer. *Int. J. Cancer*, **51,** 687–691.

CORDON-CARDO C, WARTINGER D, PETRYLAK D, DALBAGNI G, FAIR WR, FUKS Z AND REUTER VE. (1992). Altered expression of the retinoblastoma gene product: prognostic indicator in bladder cancer. *J. Natl Cancer Inst.*, **84,** 1251–1256.

CRYNS VL, THOR A, XU H-J, HU S-X, WIERMAN ME, VICKERY JR AL, BENEDICT WF AND ARNOLD A. (1994). Loss of the retinoblastoma tumor-suppressor gene in parathyroid carcinoma. *N. Engl. J. Med.*, **330,** 757–761.

FOX SB, PERSAD RA, ROYDS J, KORE RN, SILCOCKS PB AND COLLINS CC. (1993). p53 and c-myc expression in stage A1 prostatic adenocarcinoma: useful prognostic determinants? *J. Urol.*, **150,** 490–494.

HARBOUR JW, LAI S-L, WHANG-PENG J, GAZDAR AF, MINNA JD AND KAYE FJ. (1988). Abnormalities in structure and expression of the human retinoblastoma gene in SCLC. *Science*, **241,** 353–357.

HEIDER K-H, DÄMMRICH J, SKROCH-ANGEL P, MÜLLER-HERMELINK H-K, VOLLMERS HP, HERRLICH P AND PONTA H. (1993). Differential expression of CD44 splice variants in intestinal- and diffuse-type human gastric carcinomas and normal gastric mucosa. *Cancer Res.*, **53,** 4197–4203.

IWASAKI K, TORISU M AND FUJIMURA T. (1986). Malignant tumor and eosinophils. I. Prognostic significance in gastric cancer. *Cancer*, **58,** 1321–1327.

JASS JR. (1986). Lymphocytic infiltration and survival in rectal cancer. *J. Clin. Pathol.*, **39,** 585–589.

MITTHACHT S AND WEINBERG RA. (1991). G1/S Phosphorylation of the retinoblastoma protein is associated with an altered affinity for the nuclear compartment. *Cell*, **65,** 381–393.

NEKARDA H, SCHMITT M, ULM K, WENNINGER A, VOGELSANG H, BECKER K, RODER JD, FINK U AND SIEWERT JR. (1994). Prognostic impact of urokinase-type plasminogen activator and its inhibitor PAI-1 in completely resected gastric cancer. *Cancer Res.*, **54,** 2900–2907.

NINOMIYA I, YONEMURA Y, MATSUMOTO H, SUGIYAMA K, KAMATA T, MIWA K, MIYAZAKI I AND SHIKU H. (1991). Expression of c-myc gene product in gastric carcinoma. *Oncology*, **48,** 149–153.

PIETILÄINEN T, LIPPONEN P, AALTOMAA S, ESKELINEN M, KOSMA V-M, SYRJÄNEN K. (1995). Expression of retinoblastoma gene protein (Rb) in breast cancer as related to established prognostic factors and survival. *Eur. J. Cancer*, **31A,** 329–333.

PRETLOW TP, KEITH EF, CRYAR AK, BARTOLUCCI AA, PITTS AM, PRETLOW II TG, KIMBALL PM AND BOOHAKER EA. (1983). Eosinophil infiltration of human colonic carcinomas as a prognostic indicator. *Cancer Res.*, **43,** 2997–3000.

WANEBO HJ, KENNEDY BJ, CHMIEL J, STEELE JR G, WINCHESTER D AND OSTEEN R. (1993). Cancer of the stomach. A patient care study by the American College of Surgeons. *Ann. Surg.*, **218,** 583–592.

WYLLIE AH. (1993). Apoptosis (The 1992 Frank Rose Memorial Lecture). *Br. J. Cancer*, **67**, 205–208.

XU H-J, HU S-X AND BENEDICT WF. (1991). Lack of nuclear Rb protein staining in G0/middle G1 cells: correlation to changes in total Rb protein level. *Oncogene*, **6**, 1139–1146.

YU CC-W, LEVISON DA, DUNN JA, WARD LC, DEMONAKOU M, ALLUM WH AND HALLISEY MT. (1995). Pathological prognostic factors in the second British Stomach Cancer Group trial of adjuvant therapy in resectable gastric cancer. *Br. J. Cancer*, **71**, 1106–1110.